# Attitude towards psychological help in anxiety disorders

**DOI:** 10.1192/j.eurpsy.2021.507

**Published:** 2021-08-13

**Authors:** M. Vinogradova, A. Ermusheva, A. Kiselnikov, V. Tsvetkov

**Affiliations:** 1 Department Of Neuro- And Pathopsychology, Faculty Of Psychology, Lomonosov Moscow State University, Moscow, Russian Federation; 2 Department Of Pedagogy And Medical Psychology, I.M. Sechenov First Moscow State Medical University (Sechenov University), Moscow, Russian Federation; 3 Department Of Psychophysiology, Faculty Of Psychology, Lomonosov Moscow State University, Moscow, Russian Federation

**Keywords:** anxiety disorders, psychological help

## Abstract

**Introduction:**

In anxiety disorders with a lot of research on the effectiveness of treatment procedure it is important to consider patients’ implicit attitude towards mental health services, especially psychological help.

**Objectives:**

To investigate the attitude towards psychological help in anxiety disorders.

**Methods:**

In order to reconstruct an implicit attitude towards psychological help the method of color-emotional semantic associations (Kiselnikov et al., 2014) was used. Ten patients with anxiety disorders and 25 subjects from control group with no history of attending mental health services evaluated subjective differences between 15 semantic objects, 10 basic emotions and 10 colors. Factor analysis was used.

**Results:**

The analysis revealed the two-factor structure: “Valence” and “Arousal”. The semantic object “Psychological help” got 0.92 and 0.72 as first factor loadings and 0.26 and -0.65 as second factor loadings in anxiety disorders and in control group, respectively. The comparison showed a more intense and positive attitude towards psychological help in anxiety disorders. Contrariwise, the data for other semantic objects showed the tendency of more intense and negative evaluations in the clinical group.

**Conclusions:**

In anxiety disorders a shift in the categorical structure of consciousness to more negative and intense attitudes could be associated to anxiety and threat readiness. However, the attitude towards psychological help was an exception as more intense and positive which could be considered as an important factor of the effectiveness of the treatment in anxiety disorders. The research was supported by Russian Foundation for Basic Research with the Grant 17-29-02506.

